# Politics and Insanity
*"Influence des Evénemens et des Commotions Politiques sur le Développement de la Folie. Par le Docteur Belhomme. Paris, 1849."


**Published:** 1850-01-01

**Authors:** 


### Art. IV.—POLITICS AND INSANITY.*

This pamphlet is a reprint of a communication read before the *Académie de Médecine,* on May 2nd, 1848, which was noticed in No. 3 of this Journal, containing in addition the report on the paper, and the discussion which it originated.

Dr. Belhomme is the well-known director of an excellent private lunatic asylum in Paris, which has afforded him the materials of this paper, as well as two previous articles on the same subject. The first contained the particulars of some cases admitted into his establishment shortly after the Revolution of 1830; the second, of cases occurring during the stormy period which followed the accession of Louis Philippe; the present memoir contains notices of eleven cases placed under his care immediately after the Revolution of 1848. Dr. Belhomme's intention is to show that popular commotions give rise to numerous cases of insanity—that revolutionary excitement may rightly be reckoned among the moral causes of the disease; and he adduces these instances from his own practice in support of his opinion. Our readers are aware that the Académie de Médecine is in the habit of appointing a commission to inquire into the merits of the communications made to it: the commissioners chosen to inquire into Dr. Belhomme's paper, were MM. Falret, Ferrus, and Londe. The following is the substance of their report:—

"This work comprises ten reported cases of madness coming on after the events of February. The subjects were patients in the establishment of M. Belhomme. All were strongly predisposed to insanity, and five among them had been mad before the month of February; the revolution then, as far as relates to these ten individuals, was, as M. Belhomme well remarks, merely the occasional cause of insanity. The treatment employed by M. Belhomme, commonly crowned with success in a short time, has been no other than that of which experience has sanctioned the efficacy: sedatives, (and by this word we are far from meaning narcotics,) cutaneous and intestinal revulsives and distractions," &c.

A discussion followed the reading of this report, in which M. Baillarger made the following observations:—

"I think it desirable to indicate to the Academy the number of patients admitted during the year 1848, into the Hospitals Salpêtrière and Bicêtre, and to compare it with the number of patients received in the preceding years—here are the results. In 1848 there were admitted into the Salpêtrière and Bicêtre 1354 patients. The admissions in 1847 were only 1220, which gives the striking

* "Influence des Evénemens et des Commotions Politiques sur le Développement de la Folie. Par le Docteur Belhomme. Paris, 1849."

increase of 134 cases in 1848. Still we must be careful to deduce nothing from this fact, for if we look back to the five preceding years, we shall find in fact that the admissions in 1845 were 1335, and 1331 in 1846, which gives the trifling difference of only 19 to 23 more admissions for 1848. And even this slight increase is merely apparent, for loss of fortune has compelled very many families to remove their relatives from private establishments, and to have them transferred to the Salpêtrière. From the Charenton establishment alone thirty-two patients have been moved to the public hospitals, in consequence of the inability of their friends to continue the payment for their support. We may affirm, then, that the number of admissions at the Salpêtrière and Bicêtre in 1848, would have been *below* those of 1845 and 1846, but for the exceptional cause just mentioned.

"I am certainly far from asserting that *political events do not give rise to a certain number of cases of madness*, for that would be denying *evidence;* but this number seems to me much *less considerable* than is generally supposed. The publicity which attends the outbreak of insanity in notable persons during revolutionary times, has contributed not a little to the belief in a kind of epidemic. I will add but one more reflection, which is, that if political disturbances bring with them real and powerful causes of insanity, yet that they suspend other influences, which in calm and prosperous times often produce the disorder. How many passions, even in the bosom of a family, slowly sap the intelligence, and to which political events afford a happy diversion. I do not know that the two kinds of causes which I have mentioned compensate each other, and I confine myself to pointing them out as elements to be considered in the solution of the question."

M. Ferrus added—

"If there is an augmentation in the number of the insane, in some particular parts, I must declare that this augmentation is not general, and that I have not remarked it in the visitation which I have just made."

The contradictory opinion of Dr. Baillarger draws from Dr. Belhomme the following reflections:—

"What did I present to the Academy? A memoir, accompanied with cases, and I advance that, at different revolutionary epochs, I have received a certain number of mad persons, and particularly in February, 1848, a revolution attended by such sudden and crushing consequences: here I think are incontestable facts! Each commotion, each *émeute* in Paris, gave me one or more insane persons—this is clear! I have mentioned that these patients had already themselves been insane, or had had insane persons in their family. Another unequivocal remark! The madness which develops itself in these unfortunate circumstances is of the *acute* form, and is commonly easily cured.

"I am unwilling to contest the ingenious idea of M. Baillarger, but I believe that there are more causes of cerebral excitement during revolutions, than during the happy times of a wise government. The establishment of a republic gives birth to new passions; men's minds are strongly moved by a multitude of ideas and propositions more or less exciting. Another cause, not less dangerous for the ideas and the passions, are the clubs and political meetings, and the journals which breathe discord and civil war. In these critical circumstances, are there not, I ask, incessant causes of mental exaltation, and from the exaltation of the mind to its alienation there is but a step! Let us then say it, and repeat it—*political commotions and events are a powerful determining cause of madness.*"

The preceding extracts establish how great a difference in opinion exists among our Parisian brethren, upon what seems, at first sight, to be a very simple question. Do political revolutions produce insanity, or do they not? Popular commotions, do they, or do they not, tend to augment the number of insane persons? Dr. Belhomme answers in the affirmative, and we will state the general considerations which induce us to agree with our author in his conclusion.

It is a rational opinion, which we believe is confirmed by statistics, that a strong and orderly government is conducive to the mental well-being of the citizens who live under its authority. Times of licence and anarchy may perhaps be favourable to the display of individual genius, but the steady development of common talent takes place only amidst order and tranquillity. A certain amount of bodily ease and mental composure is required by the majority of mankind, and without it there can be no application, and no real attainment. Change and uncertainty are inimical to all pursuits which require slow and assiduous labour; for repose is so pleasing to our nature, that no man would voluntarily persist in working, unless there existed a reasonable prospect of his ultimately obtaining the reward of his perseverance. Firmly constituted governments, in which personal freedom is secure and property respected, guarantee to their citizens the safe enjoyment of the fruits of their exertions, be they wealth, station, or honours; but revolutionary States afford no such assurance; in them all is uncertain and insecure. Now as it is generally admitted that regular occupation and rational hope are the best preservatives of soundness of mind, it seems to us that a condition of things which precludes these cannot fail to be injurious to sanity. The quiet course of public affairs, the orderly progression of political events, and the tranquillity of social life, all exercise a beneficial influence on the mind. The calm and order of the outer world impress a peaceful image on the plastic soul. This is the link of sympathy, which, in the language of the schoolmen, connects and harmonizes the Microcosm with the Macrocosm, man's inner world with the world without. Moreover, man is essentially imitative. In a well-governed community he soon loses all disposition to act as an individual, and conforms to the common mode and habits of life. He voluntarily resigns a part of his distinctive character to assimilate and associate with his fellow-men. He lays aside his personality to obtain admission into society, and endeavours to conform with its customs and its laws. As a rational being he observed those customs and laws, and is governed and judged by them, so that any perverse departure from them is deemed a want of reason and evidence of unsound mind. A charitable, enlightened, and consistent social code would be the most efficacious check on individual aberration, but we are of opinion that the present laws of society require revision on many important points.

In every grade of life, in all countries, and at all times, there exists a certain proportion of changeful, fitful, unsettled minds which lead the unhappy possessors to afflict their friends, and astonish or amuse their acquaintance, by the strangeness of their conduct. Such persons have commonly an exalted sense of their own importance; filled with an idea of their superiority, they disregard the common opinion of mankind, and consider themselves privileged to commit any eccentricity or absurdity. Society must bow to them, not they to society. Each is the self-deified Olympian of his petty sphere. In childhood such individuals are often distinguished by their vivacity and precocious talents. In youth their cleverness continues: they are quick in apprehension, and ready in acquiring knowledge, excelling in those pursuits which please the senses, or delight the fancy; but they cannot, as a rule, master the exact sciences, or matters which require a continuous effort of thought. Arrived at manhood they exhibit something strange, irregular aud abnormal in their mode of life; they become gay, riotous, and dissipated, or misanthropic and reserved. They turn scribblers, amateur artists, schemers, political adventurers, constantly engaging in some fresh pursuit, and eager after every novelty. Destitute of any sure principle of action, without judgment, firmness, or perseverance, these unfortunate individuals fritter away life in idle schemes and fruitless speculations.

They are the high priests of quackery, of quackery political, commercial, literary, or scientific—moral or physical quacks, they become self-styled curers of men, or would-be curers of humanity.

"To such quick spirits quiet is as hell;" they abhor tranquillity —their element is confusion—they rejoice, therefore, in popular commotions, which afford the excitement they desire. These are the agitators and chief actors in revolutions, and so long as they continue in action, their minds may perhaps maintain a fictitious equilibrium; but the first interval of repose is dangerous to them, for their unstable reason is very likely to give way in the collapse which ensues. Sometimes, however, the excitement itself is fatal to them—they perish in the storm they have helped to raise; for, as Dr. Belhomme observes, "There is but a step from exaltation of mind to alienation."

But in all revolutions the number of actors is extremely limited—the majority of the nation is content to suffer. The strongly-expressed will of a few noisy men, banded together for mischief, is sufficient to coerce the weak and faltering wishes of the patient mass of the community. In such times the active spirit of evil is infinitely more potent than the passive principle of good. For one person who immediately profits by a revolution there are hundreds who suffer; and we see in this fact a fertile cause for the increase of insanity. Nearly all writers on the subject agree in ascribing a greater influence to the moral than to the physical causes of the disease. Pinel computed that the moral causes rather more than doubled the physical: Esquirol, that the moral causes are to the physical as 4 to 1. The nature of the subject does not admit of very precise statistics. For instance, we think it erroneous to class in all cases "hereditary predisposition" among the physical causes, since the actual manifestation of insanity in persons hereditarily predisposed is frequently induced by a moral cause, under the influence of which they become mad, but exempt from which they might have remained sane for life. However, let the exact proportions be what they may, it is an established fact, that the moral causes of insanity greatly exceed the physical. Now what are the moral causes? We will take Esquirol's classification.

"Domestic grief—disappointed love—political events—fanaticism—fright—jealousy—anger—poverty, reverses of fortune—wounded self-love—disappointed ambition—excess in study—misanthropy."

In perusing this list do we not seem to enumerate most of the evils which inevitably accompany a subversive revolution?

Indeed, the disasters which attend the downfal of governments present so many mournful causes for the overthrow of minds, that we are surprised to find Dr. Baillarger denying their influence in increasing the number of the insane, and are ready to decide with Dr. Belhomme, that political events and public commotions are powerful determining causes of madness. The fact that ten cases of mania, all produced by the events of February, were brought to Dr. Belhomme's private establishment, is in itself strong evidence in favour of the correctness of his conclusion. For if ten cases fell to the share of one asylum, the total increase must have been very considerable. We do not think the argument of Dr. Baillarger conclusive—viz., that the number of patients in the great public hospitals was not augmented by the revolution. Patients admitted into charitable asylums belong almost exclusively to the poorer classes, and it is not on them that the first shock of a revolution falls. To the mass of the community, literally condemned to daily labour for their daily bread, any change is welcome, and they favour universal commotion as a means of improving a position, which they fancy cannot be rendered worse. To them revolutions inspire more hope than anxiety. This was peculiarly the case with the last French revolution, which at once assumed a Communist character, and which was styled by the popular press, the "revolution of the working classes." Every man of the 200,000 "ouvriers" who defiled before the Hotel de Mille, on the 16th of March, saw in the revolution a cause for rejoicing; but how opposite were the feelings of the timid citizens, when they viewed that monster procession passing before their windows. Without was noise and exultation, within was misgiving and dread. The revolutionary hydra was roaring in the streets, and no one knew who might be its victim. "Care and anxiety, distress, grief, and mental disturbances, are by far the most productive causes of insanity. These causes are at all times influential in civilized countries, and hence one principal reason why insanity prevails in proportion to the cultivation of society."* And not only is insanity more frequent in civilized than in barbarous countries, but in the most civilized state it is the most highly-educated class which affords the largest contingent of sufferers. In proportion as the standard of mental culture and intellectual refinement is exalted, so much the greater becomes the liability to a departure from it.

It follows from this, that the ill-informed masses of the people are not so likely as the educated to be affected by the outbreak of a revolution—untaught and unteachable by experience, ignorant of past miseries, filled with false expectations of the future, they embrace any change with hopefulness. The page of history is a blank to them; they know neither its lessons nor its warnings; they see not the uselessness of popular tumult, the errors of popular prejudices, the fallacies of popular belief. They see but one thing—the chance of ob-

* Prichard. "A Treatise on Insanity," p. 183.

taining their living without working for it. When, therefore, we learn from Dr. Baillarger that the revolution of February did not materially increase the number of admissions into the state pauper asylums, we are by no means astonished; nor do we think Dr. Belhomme's conclusions, drawn from private practice, at all invalidated by that fact. There is also another circumstance which would reduce the number of applications for pauper lunatics,—namely, that in times of great social disturbance very many cases of insanity amongst the people pass unnoticed. When all the world is mad, individual derangement fails to attract attention. How many madmen roamed at large in France between February and May, 1848, haranguing, denouncing, and propagandizing? How many fell behind the barricades in June? How many found their way into a prison instead of an hospital? How many carried their lunacy into other lands? The eloquent memoir of the lamented Pariset, quoted by Dr. Belhomme, contains some remarks applicable to the point; and although a translation of some portion of it is given in Dr. Prichard's "Treatise on Insanity," we feel assured that our readers will excuse our reproducing it.

"During the great tumult of revolutions, while all the social elements were convulsed in every way, some thousands of cases of derangement probably took place. Reverses of fortune, sudden changes which exalted and humbled individuals, so many sources of disaster opened at the same time, overwhelmed France with unexampled calamities; cases of madness doubtlessly appeared in considerable numbers, *but were lost sight of in the currents of great events.* Moreover, those who know to what a degree the habits, the disorders, the infirmities, of mind as well as body are transmissible, will not think it rash to conclude that even the children begotten at that epoch, experienced its baneful influence in their mothers' wombs. In general, every great and rapid change, be it in the physical or the moral order or things, is pernicious to the health and to the reason. The sight of greatness overthrown, and equal greatness acquired by strokes of fortune altogether unexpected, did not simply excite astonishment, but gave rise, even in the rudest minds, to most dangerous hopes and illusions. Universal reformers, founders of empires and republics, concoctors of constitutions, showed themselves on every side; simple artisans, and even labourers, thought themselves destined to ascend a throne. These wild fancies, so flattering to self-love, are unfortunately the most obstinate of aberrations; for of all the kinds of derangement those sprung from pride present the greatest resistance; they are so suspicious and so irritable that they array themselves against all who caress them, and are for that reason incurable. I may add, that they are very numerous, for it would appear that the diabolical pleasure of ruling over men is the most exquisite delight of the human race."

With the foregoing quotation we conclude our observations upon this part of the subject. On analyzing Dr. Belhomme's eleven cases, we find that they all presented the character of *acute mania,* attended, in six cases, with violent raving. In addition to his own eleven cases, the author gives one similar case, communicated by Mr. Jolly, also acute mania. Eight or nine patients, received under similar circumstances in 1830—1832, were all affected with acute mania. We may conclude, therefore, that mania is the form of insanity most commonly developed under circumstances of strong sudden excitement. Of the eleven cases, six were clearly produced by fright, three by the contagion of popular tumult, and two by political exaltation. The cases caused by fright were the most violent; indeed, fright seems almost invariably to produce the maniacal form of the disease. Esquirol mentions that thirty-six out of thirty-eight cases of madness produced by fright were instances of mania. Theoretically, one might fancy that insanity following fright would assume the form of "*deomania,*" or fear-madness, but such is not the case. And this agrees with the observation of Pinel, who says—

"To believe that the different species of insanity depend on the particular nature of its causes, and that it becomes periodical, continued or melancholic, according as it may have originated from unfortunate love, domestic distress, fanaticism, superstition, *or interesting revolutions in the state of public affairs,* would be to fall into a very great error. My experience authorizes me to affirm that there is no necessary connexion between the specific character of insanity, and the nature of its exciting cause."

That so large a proportion as six out of eleven cases of mania should have been produced by fear, is certainly a remarkable fact, which we are inclined to ascribe in some extent to the terrorizing character of the recent periodical literature in France.

During the last ten years of the reign of Louis Philippe, the leading French romancers appeared to have entered into a secret compact to revile the character of their nation, and to render their beautiful capital a plague spot, and an abomination in the eyes of Europe. If their revelation was to be credited, Paris was a vast den of rapine, lust, and murder; in which it was stark folly for any one who valued existence to reside. De Balzac, Soulie, Eugène Sue, Alexandre Dumas, Paul Féval, and even the old thief-catcher, Vidocq, in turn unveiled some hideous feature in the mysteries of crime. They told their credulous fellow citizens that there were living within their walls, many thousands of monsters, human only in shape, to whom blood was as water, man's life a jest, and mortal agony a sport. Nor was the mania of revilement confined to the romance writers; even grave staticians caught the infection, and published a supposititious census of the throat-cutting class of the community. The so-called historians followed next, ransacking the barbarous chronicles of the middle ages for instances of the horrible. Finally came the world-famous "History of the Girondists," and set the seal on this terrible catalogue.

When, therefore, after the brief conflicts on the 24th of February, the populace triumphed, and the mob was master of Paris—when law had ceased to exist, and universal anarchy prevailed—many thousands of the inhabitants expected an immediate renewal of the awful scenes which had characterized the first revolution. Apprehension seized on the minds even of men, but more especially on the minds of the women, many of whom were left alone in their houses; their husbands, brothers, or sons, being still in the streets. They had been daily taught for years, by the public press, that their city was filled with demons, whose evil disposition was with difficulty restrained by the armed power of the law; and, consequently, the intelligence that all restraint was removed, filled them with terror and dismay, and left them a prey to the constant dread of massacre and pillage. Fright was caused also in another way—by the indescribable alarm which assails the breast when the rattle of musketry, and the roar of cannon, announce that the bloody work of slaughter is going on in the midst of a populous city. Such sounds, whose effect only those who have heard can conceive, produce an emotion which, for a moment, blanches the cheek, and stays the breath of the bravest. From such events, and under such circumstances, we need not wonder that many timid and excitable persons, of both sexes, lost their reason. The result of the revolution showed how deeply the "*feuilletonistes*" had calumniated the lower order of their fellow-citizens, for we do not remember to have heard, and we were present, any one instance in Paris, in which a private person was ill-used, or private property attacked. But the truth was revealed too late, the mischief was already done, and the register of Dr. Belhomme's establishment bore witness to the calamitous influence exercised by the writings of the sensation-mongers.

Our author's method of treatment is certainly more active than that which we usually follow in this country, consisting in bleeding, leeching, cupping, purging, prolonged bathing, cold effusions on the head, and the administration of sedatives: however, it seems to have been eminently successful.

In conclusion, we have to offer our best thanks to Dr. Belhomme, for his interesting memoir; and trusting that his professional brethren, connected with similar establishments, public and private, may be induced to follow his example, we hope to have some additional information on this interesting subject to communicate in a future number.

---

